# Isolated dentinogenesis imperfecta: Novel *DSPP* variants and insights on genetic counselling

**DOI:** 10.1007/s00784-024-05636-z

**Published:** 2024-04-17

**Authors:** Nehal F. Hassib, Mennat Mehrez, Mostafa I. Mostafa, Mohamed S. Abdel-Hamid

**Affiliations:** 1 Orodental Genetics Department, Human Genetics and Genome Research Institute, National Research Centre, 33 ElBohous street, Dokki, P.O.12622, Cairo, 3337 09 31 Egypt; 2 Medical Molecular Genetics Department, Human Genetics and Genome Research Institute, National Research Centre, Cairo, Egypt

**Keywords:** Dentinogenesis imperfecta, *DSPP*, Novel variants, Genetic counseling, Egyptian patients

## Abstract

**Objective:**

Dentinogenesis imperfecta (DI) is an inherited dentin defect and may be isolated or associated with disorders such as osteogenesis imperfecta, odontochondrodysplasia Ehler-Danlos and others. Isolated DI is caused mainly by pathogenic variants in *DSPP* gene and around 50 different variants have been described in this gene. Herein, we report on 19 patients from two unrelated Egyptian families with isolated DI. Additionally, we focused on genetic counselling of the two families.

**Materials and methods:**

The patients were examined clinically and dentally. Panoramic X-rays were done to some patients. Whole exome sequencing (WES) and Sanger sequencing were used.

**Results:**

WES revealed two new nonsense variants in *DSPP* gene, c.288T > A (p.Tyr96Ter) and c.255G > A (p.Trp85Ter). Segregation analysis by Sanger sequencing confirmed the presence of the first variant in all affected members of Family 1 while the second variant was confirmed to be *de novo* in the patient of Family 2.

**Conclusions and clinical relevance:**

Our study extends the number of *DSPP* pathogenic variants and strengthens the fact that *DSPP* is the most common DI causative gene irrespective of patients’ ethnicity. In addition, we provide insights on genetic counseling issues in patients with inherited *DSPP* variants taking into consideration the variable religion, culture and laws in our society.

**Supplementary Information:**

The online version contains supplementary material available at 10.1007/s00784-024-05636-z.

## Introduction

Odontoblasts, derived from the ectomesenchyme, are responsible for the formation of dentin. Once fully differentiated, they begin to deposit the organic dentin matrix rich in collagenous and non-collagenous proteins, a process that ends with the mineralization of the pool of dentin matrix excreted by the odontoblasts [1].

On the molecular level, around 300 genes involved in the formation of the normal hard dentin in odontoblasts have been identified. Of them, some are responsible for extracellular matrix (ECM) deposition, others are for mineralization, while certain genes are responsible for the signaling pathways. There are also genes that regulate innervation and aging. However, the specific role of many of these genes remains unclear. Mutations in any of the ECM genes result in the disruption of the protein function, and abnormal dentin formation in both permanent and deciduous dentitions [2].

Dentin sialophosphoprotein (DSPP) and dentin matrix acidic phosphoprotein 1 (DMP1) form the non-collagenous network protein matrix which is considered the niche of calcification. DSPP is secreted at high levels in fully differentiated odontoblasts, unlike DMP1 which is also found in osteoblasts, making DSPP an indicator for odontoblast maturation [3].

Pathogenic variants in *DSPP* are associated with three non-syndromic autosomal dominant dentin defects, dentin dysplasia type II (OMIM# 125,420), dentinogenesis imperfecta (DI) Shields type II, III (OMIM# 125,490, 125,500) and non-syndromic hearing loss with DI (OMIM# 605,594) disorders. The associated phenotype includes dentin dysplasia, opalescent/amber teeth, thin short roots, and wide or obliterated pulp chambers [4]. To date, around 50 different pathogenic *DSPP* variants have been described in the literature. These variants are mostly protein truncating or missense with few reported splice variants and inframe deletions. Majority of variants are clustered in exon 5 of the gene while only one variant (p.Arg65Trp) have been described in exon 4 [5].

This study reports the oro-dental and molecular findings of 19 patients (a large pedigree with 18 affected individuals and a sporadic patient) with isolated dentinogenesis imperfecta. Additionally, we provide insights on genetic counselling of patients especially those with familial DI in accordance with the variable beliefs regarding religion, culture and law in our society.

## Materials and methods

### Patients

Nineteen patients from two unrelated families were recruited from the outpatient clinic of Oro-Dental Genetics Department, Centre of Excellence, National Research Centre (NRC), Cairo, Egypt. The chief complaint of the affected individuals was abnormal friable discolored teeth. The study design was explained to the patients and their parents. The study was revised and approved by the Oro-dental Genetics Reviewer Scientific Board of the Department and the Medical Research Ethics Committee of NRC (Approval: 20068), Cairo, Egypt. The patients/guardians signed a written informed consent to accept the dissemination of the data. Patients were subjected to meticulous clinical and dental examinations and photo and pedigrees were taken as well.

### Methods

Genomic DNA was extracted from peripheral blood samples of all affected patients and their parents using Qiagen Blood DNA Kit (Qiagen, Hilden, Germany). Whole exome sequencing (WES) was performed for one patient from each family (Patients 13 and 19) using SureSelect Human All Exome 50 Mb Kit (Agilent, Santa Clara, CA, USA) and analyzed on Illumina NovaSeq 6000 (Illumina, San Diego, CA, USA). The obtained sequences were aligned to UCSC human genome GRCh37/hg19 and variants were verified through the GATK pipeline. Identified variants were checked against public genetic databases like Genome Aggregation Database (gnomAD, https://gnomad.broadinstitute.org/), 1000 Genomes (www.1000genomes.org), and dbSNP (http://www.ncbi.nlm.nih.gov/SNP/). Pathogenicity of detected variants were predicted using different bioinformatics tools as MutationTaster (https://www.mutationtaster.org/), SIFT (https://provean.jcvi.org/protein) and PolyPhen-2 (https://genetics.bwh.harvard.edu/pph2/). In addition, the NMDEscPredictor tool was used to assess the impact of the identified variants, whether they escape or do not escape nonsense mediated decay (https://nmdprediction.shinyapps.io/nmdescpredictor/).

### Segregation analysis

Segregation analysis of the identified variants in the parents and other affected family members of Family 1 was conducted by PCR amplification of exon 4 of the *DSPP* using specific primer designed by Primer3 software followed by purification with Exo-SAP PCR Clean-up kit (Fermentas, Germany) and sequencing using the BigDye Terminator v3.1 Cycle Sequencing Kit (Applied Biosystems, Foster City, CA, USA) on the ABI Prism 3500 Genetic Analyzer (Applied Biosystems) according to manufacturer’s instructions.

## Results

### Clinical and oro-dental data

The clinical and the radiological examinations of the 19 patients excluded the presence of fractures, bone aches or hearing defect and therefore they were classified as “isolated dentinogenesis imperfecta”.

#### Family 1

**Fig. 1:**
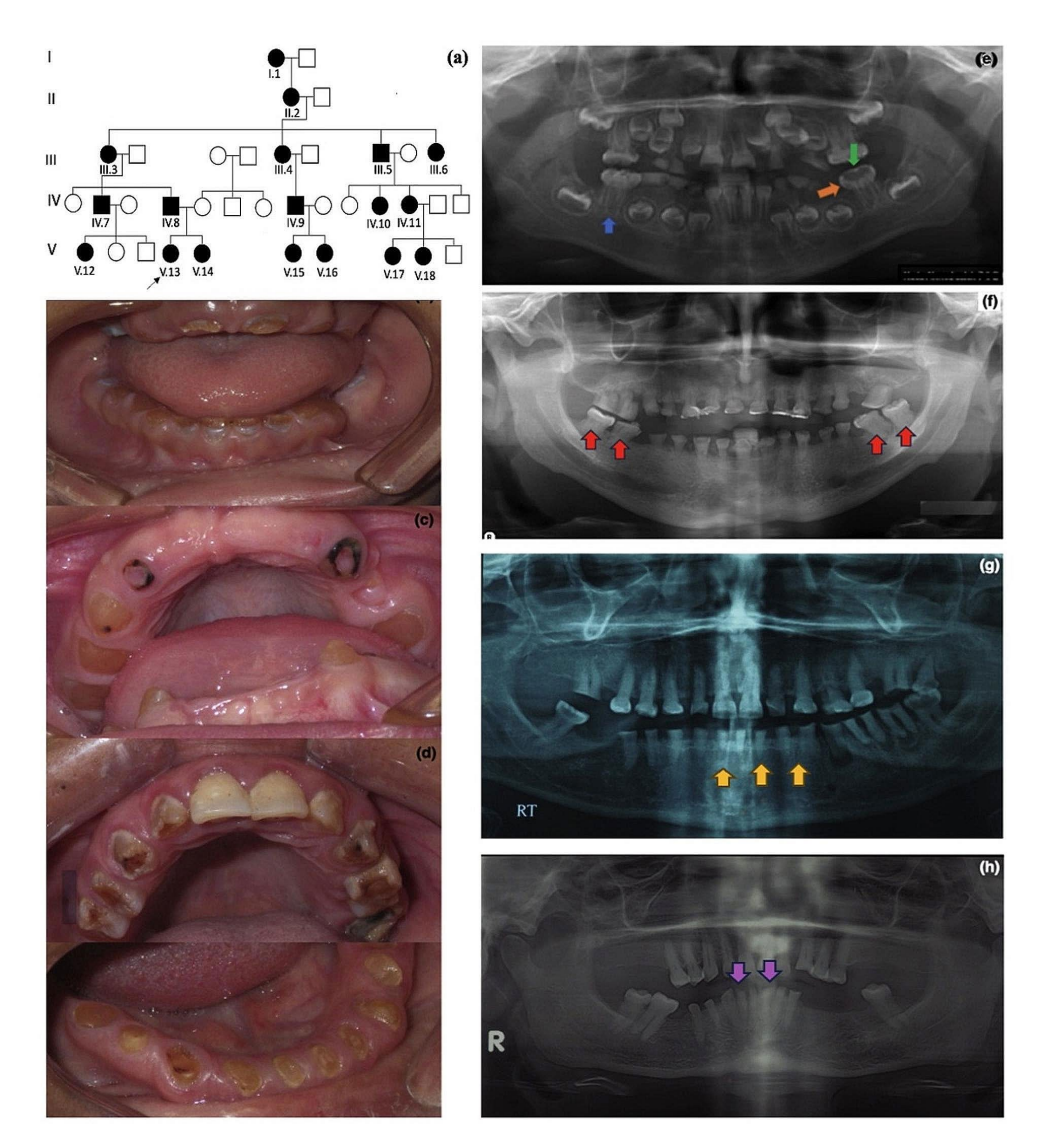
(**a**) Pedigree of Family 1 showing no consanguineous marriage, and the large number of affected members (**b**), (**c**), (**d**) Intraoral photos of Patients 13, 14 and 4 showing full set of dentitions with dentinogenesis imperfecta, the lost teeth in figure c were extracted due to extensive caries. (**e**) Panoramic view of Patient 13 showing thin root (blue arrow) with loss of dentin layer and wide pulp chamber (green arrow), bulbous crowns (orange arrow). (**f**) Panoramic view of Patient 9 showing obliterated pulp chamber (red arrows). (**g**) Panorama of Patient 4 showing short roots (yellow arrows) of lower anterior teeth. (**h**) Panorama of Patient 3 showing microdontia of lower affected anterior teeth (pink arrows)

Family 1 is a large pedigree comprising 18 affected individuals (Patients 1–18). They were 14 females, and 4 males and their age ranged from 6 to 85 years old (Fig. 1a). Only five patients were participated in the clinical examination (two siblings (Patients 3, 4, 9, 13, and 14). The other family members provided only the blood samples. Transportation from Upper Egypt and investigations’ costs prohibited the remaining family members to achieve the full clinical examination requested. The teeth were amber in color with chipped enamel and teeth sensitivity in all except one (Patient 9). Both dentitions in younger patients had the same phenotype (Fig. 1b-c**)**. The intraoral examination of adult patients displayed severely attritted affected dentitions (Fig. 1d**)**. Panoramic view revealed thin roots with a thin dentin layer and wide pulp chambers. Bulbous crowns of molar teeth were observed in the radiograph. (Fig. 1e). The patient (Patient 9) with no pain or sensitivity had complete obliteration of the pulp which explained his symptoms (Fig. 1f). Short roots (P4) and microdontia (P3) were also seen in some examined patients (Fig. 1g, h).

#### Family 2

**Fig. 2:**
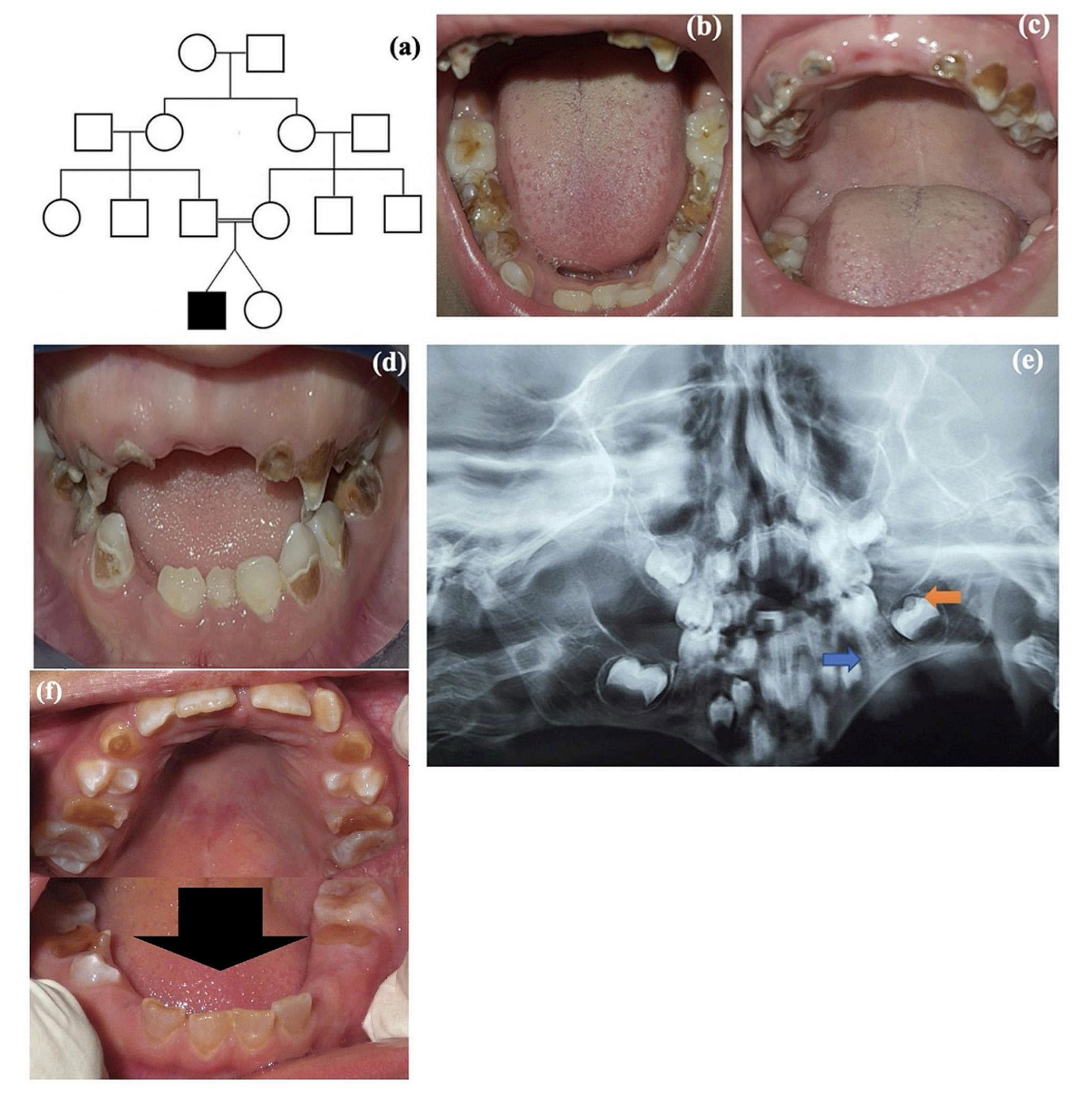
(**a**) Pedigree of Family 2 with positive consanguinity (**b-d**) Intraoral photos showing opalescent lower permanent anterior teeth, amber colored upper and lower deciduous teeth, (**e**) panorama showing thin roots (blue arrow), wide pulp chamber (orange arrow) and bulbous crowns of molar teeth. ((**f**) Intraoral photo at the age of 9 years and 6 months showing dentinogenesis imperfecta in certain dentitions mainly in lower anterior teeth (black arrow)

Patient 19 is a 7-years-old boy born to healthy consanguineous parents and was a twin of a normal female sib (Fig. 2a). Dental examination revealed opalescent teeth in association with destructed dentition. The dentin defect was not present in the full set of teeth due to the badly teeth destruction which avoided the proper color evaluation. It was present mainly in the deciduous canines, 1st deciduous molars and slightly on the permanent lower anterior incisors. Moreover, a high arched palate, long uvula and median grooved tongue were detected (Fig. 2b-d). Due to the short attention of the child, a panoramic radiograph of the teeth was taken with distortion, however, it exhibited thin roots, wide pulp chambers and bulbous crowns of the molar teeth. The dentin layer was not well demarcated in the panoramic film (Fig. 2e**)**. The patient was uncooperative, periapical x-ray could not be taken, as a result we moved to panoramic alternative. At the age 9 years and 6 months, the patient was recalled and his oral cavity was examined. Some permanent dentitions were erupted with dentinogenesis imperfecta affecting mainly the lower anterior incisors (Fig. 2f**).** The patient was continuously uncooperative which did not enable us to have new dental x-rays.

**Fig. 3:**
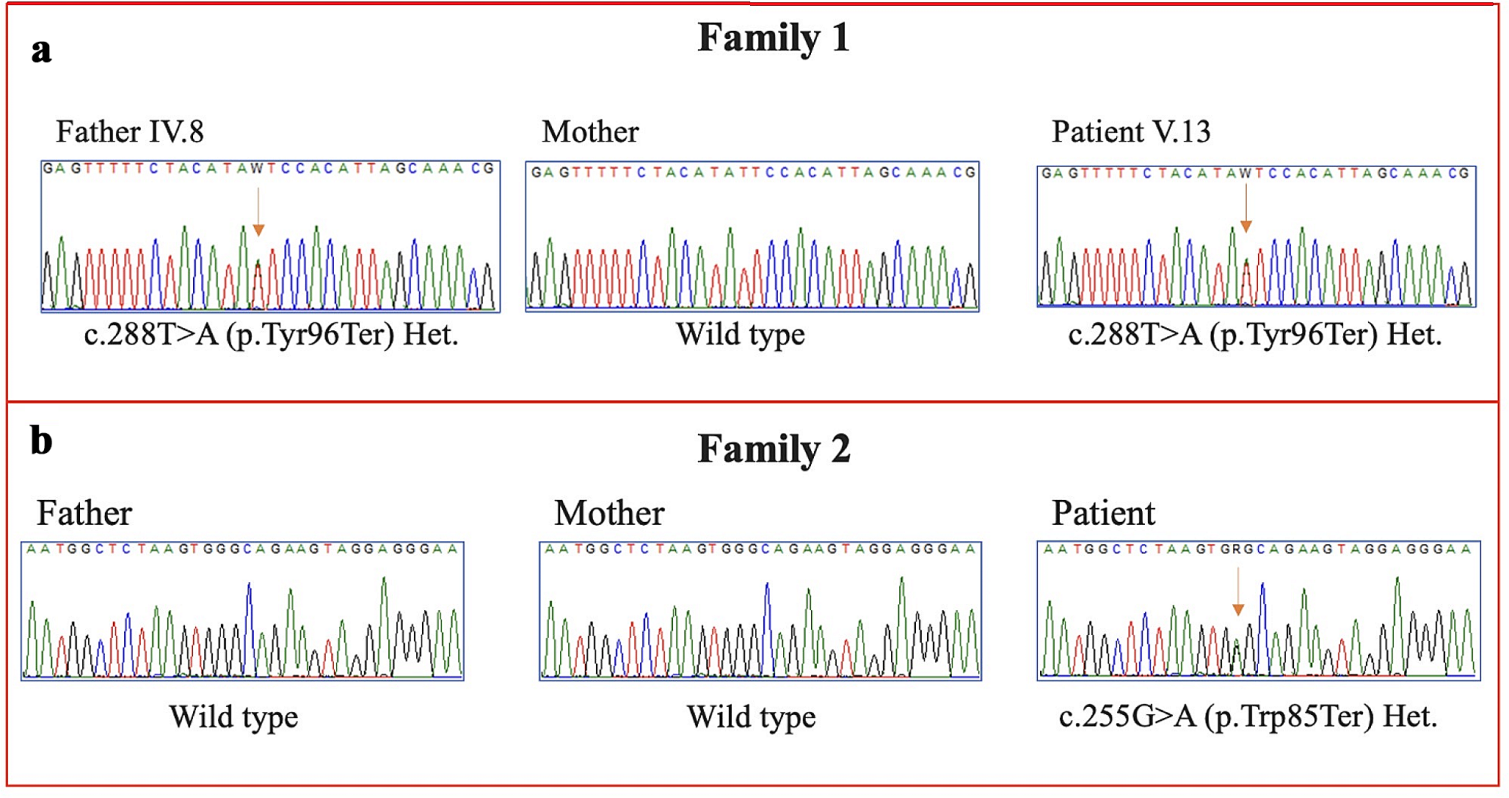
Part of the sequencing electropherograms showing the *DSPP* variants identified in our study. The arrow indicates the site of the variant

### Molecular findings

WES analysis of Patient 13 (Family 1) revealed a new heterozygous nonsense variant in *DSPP* gene, c.288T > A (p.Tyr96Ter). *DSPP* gene is associated with dentinogenesis imperfecta type II. Segregation analysis confirmed the presence of the variant in all affected individuals (Fig. 3a and Supplementary Fig. 1). The single patient of Family 2 (Patient 19) was also found to harbor a heterozygous nonsense variant (c.255G > A, p.Trp85Ter) in *DSPP*. The c.255G > A (p.Trp85Ter) variant was confirmed to be *de novo* as both parents were wild type (Fig. 3b). The two *DSPP* variants were not found in public genetic databases or our-inhouse database of more than 1500 exomes. In addition, they were predicted to be deleterious by different bioinformatic tools. According to the American College of Medical Genetics (ACMG), the two variants should be classified as “likely pathogenic”.

### Genetic counseling

Counseling was offered to each family independently explaining the disorder and its inheritance. Management and rehabilitation methods were explained according to the patients’ condition and age. The possibility of having the same condition in future pregnancies was explained several times, as some counselees tend to think that if they had several children with the condition then their probability of having affected children would be decreased.

In Family 1, after the counselor made sure that this information is perfectly understood, one of the mothers reciprocated that she wanted to have normal children and asked about the means to do that. After presenting the options of pre-implantation genetic testing and in-vitro fertilization as well as amniocentesis in case they chose natural conception and abortion of the affected pregnancy, the counselor chose to advise the parents that the latter procedure which is more suitable to their socioeconomic class would be difficult to attain, due to the religious constraints in Egypt. The counselor returned and pressed on the fact that the condition is considered one of the manageable conditions and that early on the children will need psychological support to be able to handle comments from their peers until their ages are suitable for dental intervention. In Family 2, as the variant was not inherited from any of the parents, parents were reassured as the risk of having affected children in the future is minimal. Additionally, the parents were informed about the possibility of gonadal mosaicism. However, their son had a risk of 50% of having affected offspring. They promised to pass this information to their son at his appropriate age.

## Discussion

Dentin is the main hard core forming the tooth body, situated beneath enamel and cementum. It is the defender of the sensitive pulp tissue. The main component of dentin is crystalline tissue, organic matrix, and water. Collagen type I predominates in the collagenous protein network of the dentinal configuration [6] The current advancement in the field of molecular genetics allowed unraveling of the pleiotropic action of many genes involved in odontogenesis with their complicated interactive networking and pathways [7]. Dentin defect is the sequela of altered extracellular matrix formation and mineralization. Dentin dysplasia and dentinogenesis imperfecta are inherited monogenic disorders with an autosomal dominant pattern of inheritance [4].

In this study, we identified heterozygous *DSPP* variants as the cause of DI in our two families. Two new nonsense variants: c.288T > A (p.Tyr96Ter) and c.255G > A (p.Trp85Ter) in the same exon (exon 4) were identified. Both variants are expected to result in early protein truncation and nonsense mediated decay. So far, around 50 different *DSPP* variants have been described in patients with DI or dentin dysplasia from various ethnic groups [5, 8–13]. Majority of these variants are located in exon 5 which is likely due to its large size rather than being a hot-spot exon for mutations. Although several protein truncating variants have been reported in the *DSPP* gene, only one nonsense variant (c.133 C > T, p.Gln45Ter) has been previously described [14, 15]. This variant is located at the splice region of exon 3, three nucleotides before the end of the exon. Several studies have confirmed the splicing effect of this variant leading to skipping of exon 3 [16, 17]. In contrast, the identified variants in our study are located away from the splice site of exon 4 and therefore are not likely to alter splicing. As a result, both of these variants might be considered the first true nonsense variants identified in the *DSPP*. In view of reported variants, very few were recurrent e.g. the c.52G > T (p.Val18Phe) which was described in 6 unrelated families of Chinese (3 families), Korean, Caucasian, and Finnish origin [18–21, 9]. Also, the c.53T > A (p.Val18Asp) was reported in three families from Korea [22, 23] and Japan [24]. Other variants were even rarer and found in one family each.

The *DSPP* gene consists of five exons and encodes the dentin sialophosphoprotein which has two main domains, the amino-terminal dentin sialoprotein (DSP) and carboxyl-terminal dentin phosphoprotein (DPP). Exons 1 to 4 and the anterior part of exon 5 encode for the DSP while the posterior part of exon 5 encodes for DPP. Previous studies have showed that variants in the DSP region, regardless of their type (missense or protein truncating), are associated with DI while protein truncating variants in the DPP are associated with the dentin dysplasia [8, 15, 16]. Interestingly, our two families harbored variants in the DSP region and presented with DI. Therefore, our results are in accordance with such observation and support the presence of phenotype-genotype correlations in patients harboring *DSPP* variants.

Although *DSPP* gene seems to be the main causative gene for DI, some reports failed to identify variants in the gene in patients with DI [5, 9]. This led authors to suspect the presence of other etiologies for DI. Recently, heterozygous variants in *COL1A2* gene, which usually cause osteogenesis imperfecta type II, III and IV as well as several types of Ehler-Danlos syndrome, were reported in three families with non-syndromic dentinogenesis imperfecta by Lee and co-authors [23]. The study of Lee et al. broadened the spectrum of DI causative genes and highlighted the importance of using whole exome sequencing to unravel novel DI genes [23].

Dentinogenesis imperfecta is known to be associated with pulp obliteration and thin roots. The defective dentin has atypical effect towards external stimulants which might lead to hypertrophied dentin formation and pulp chamber obliteration [24]. As a result, periapical pathosis occurs. The prevalence of periapical lesions is greater in narrow root canals or obliterated. Consequently, loss of teeth due to pathosis is prominent [8]. Elder patients of Family 1 complained of complete pulp obliteration without exhibiting any periapical pathosis.

Genetic counseling and risk assessment are performed under strict ethical considerations taking into account the psychological and socioeconomic class of the counselee. The ethical considerations particularly respecting the counselee’s autonomy and privacy are considered universal that is to say they are protected at all instances regardless of the counselee’s ethnicity and religious beliefs. Over the years, the concept of the counselee’s autonomy has evolved from strict non-directiveness to balanced and well-educated protection of the counselee’s autonomy [25]. This what was followed in the sessions with our families specially Family 1.

In Muslim countries like Egypt, abortion is allowed in case the fetus is proven to be deformed to the degree that the quality of life would be diminished considerably as in multiple congenital anomalies, neurodegenerative diseases or metabolic disorders that have no cure yet among other disorders and this must be done before the end of the fourth month of pregnancy [26]. For a condition like DI where the patients’ complaint is only the yellowish color of the teeth, abortion would be legally and religiously prohibited. However, parents of Family 1, like other parents across the globe, were very concerned and wanted to have kids with normal teeth to avoid subjecting them to bullying from other kids and school colleagues. They were informed about the possibility of pre-implantation genetic (PGD) testing which would save them the hassle of prenatal testing and abortion. However, a major problem with PGD is its high cost which cannot be afforded by majority of parents in our country. Therefore, parents were advised to seek psychological help for for their affected children which is empirical in these cases [27]. On the contrary, the outcome of genetic counseling of Family 2 was very positive as parents were very happy when informed that neither of them carries the DSPP variant and that the recurrence risk has decreased from 50% in each pregnancy to less than 1–2% [28]. However, we are aware that parental gonadal mosaicism was not ruled out in this family.

## Conclusion

We reported two new families with DI from Egypt carrying novel *DSPP* variants expanding the mutational spectrum of DI and reinforcing the old notion that variants in the DSP region are associated with the DI phenotype. The collaboration between multidisciplinary genetics teams is mandatory to reach precise diagnosis. Addressing the psychological impact of genetic testing of manageable dental diseases in Muslim countries is a worthy point of future research. Further studies are also recommended in dental molecular biology and different pathways for understanding the causative genes and epigenetics influencing the odontogenesis.

## Electronic supplementary material

Below is the link to the electronic supplementary material.

Supplementary material 1
