# Supplementary material for: Isolated dentinogenesis imperfecta: Novel DSPP variants and insights on genetic counselling
Source: Clin Oral Investig. 2024 Apr 17;28(5):254. doi: 10.1007/s00784-024-05636-z (PMC11024031; doi:10.1007/s00784-024-05636-z)
Supplement: Supplementary file 1 — Supplementary material 1 [file 784_2024_5636_MOESM1_ESM.docx]

**Supplementary Figure 1**

**
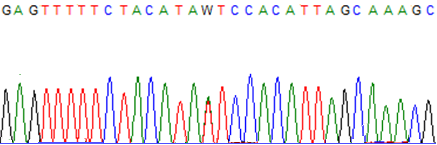
**

II.3

**Supplementary Figure 1:** Portion of the sequencing electropherograms showing the segregation of the c.288T>A (p.Tyr96Ter) variant in all affected individuals of Family 1. The arrow indicates the site of the variant.
